# The N6-Methyladenosine RNA Demethylase AlkB Homolog 5 (ALKBH5) in Metabolic Diseases: Molecular Mechanisms and Pharmacological Implications—A Review

**DOI:** 10.3390/biom16040499

**Published:** 2026-03-26

**Authors:** Guida Cai, Leyi Fu, Xi Zhang, Meiling Yan

**Affiliations:** 1 Key Laboratory of Glucolipid Metabolic Disorder, Ministry of Education of China Guangdong, Institute of Chinese Medicine, Guangdong Metabolic Diseases Research Center of Integrated Chinese and Western Medicine, Key Laboratory of Metabolic Disease Prevention and Treatment of Traditional Chinese Medicine, Guangdong Pharmaceutical University, Guangzhou 510006, China; 2 Guangdong Provincial Engineering, Center of Topical Precise Drug Delivery System, School of Pharmacy, Guangdong Pharmaceutical University, Guangzhou 510006, China

**Keywords:** metabolic diseases, ALKBH5, m^6^A RNA methylation, epitranscriptomic regulation, therapeutic potential

## Abstract

Metabolic diseases, including type 2 diabetes mellitus (T2DM) and metabolic dysfunction-associated fatty liver disease (MAFLD), are chronic disorders characterized by dysregulated glucose and lipid homeostasis and represent major contributors to insulin resistance, cardiovascular complications, and liver injury. Despite considerable progress in elucidating their pathogenesis, effective preventive and therapeutic strategies remain limited. N6-methyladenosine (m^6^A) RNA demethylase AlkB homolog 5 (ALKBH5), a nuclear epitranscriptomic “eraser,” broadly regulates post-transcriptional gene expression by modulating RNA splicing, nuclear export, stability, and translation. Dysregulation of ALKBH5 has been implicated in tumorigenesis, immune dysfunction, and stress responses, underscoring its wide-ranging biological significance. Emerging evidence further indicates that ALKBH5 plays a pivotal role in maintaining metabolic homeostasis. However, most existing reviews have focused primarily on its roles in cancer, leaving its functions in metabolic diseases relatively unexplored. In this context, this review summarizes the structural characteristics and molecular mechanisms of ALKBH5 and discusses its emerging roles across a spectrum of metabolic diseases, including MAFLD, metabolic complications such as diabetic retinopathy (DR), diabetes-associated cognitive impairment (DACI), atherosclerosis (AS), and diabetic cardiomyopathy (DCM), as well as metabolism-related inflammatory diseases represented by rheumatoid arthritis (RA). Furthermore, recent pharmacological strategies targeting ALKBH5 are discussed, with attention to the challenges posed by its context-dependent, tissue-specific, and disease stage-specific activities. Overall, ALKBH5 emerges as a key epitranscriptomic regulator in metabolic diseases, and advancing therapeutic strategies that account for molecular context and tissue specificity will be critical for achieving safe and effective clinical interventions.

## 1. Introduction

Metabolic diseases, including type 2 diabetes mellitus (T2DM), obesity, metabolic-Associated Fatty Liver Disease (MAFLD), metabolic syndrome, and related cardiovascular complications, are among the most pressing global public health challenges [1,2,3,4]. Epidemiological evidence shows that about one in ten adults worldwide currently lives with diabetes, which means that hundreds of millions of people are affected across different regions [5]. Many of these individuals are not diagnosed in time, or they do not receive sufficient treatment. At the same time, the continuous increase in obesity and MAFLD further worsens cardiovascular, renal, and neurological complications. These changes lead to higher rates of illness and death and place a growing burden on health-care systems. For this reason, it is necessary to clarify disease mechanisms, to identify biomarkers that allow early detection, and to develop therapeutic strategies that are more targeted and effective [6,7,8].

At the molecular level, N^6^-methyladenosine (m^6^A) is the most abundant internal modification in eukaryotic mRNA and noncoding RNA, and it plays a central role in RNA regulation [9,10]. This modification influences RNA splicing, nuclear export, RNA stability, translation efficiency, and RNA degradation. The m^6^A modification is dynamically regulated rather than static. This regulation depends on the coordinated actions of “writers,” such as the METTL3–METTL14 complex, “readers,” including members of the YTH and IGF2BP protein families, and “erasers,” such as FTO and ALKBH5. Through this system, RNA fate can be adjusted in a reversible and controlled manner. Accumulating studies indicate that m^6^A modification and its regulatory proteins are closely involved in developmental processes, maintenance of metabolic homeostasis, and the onset and progression of disease [11,12,13,14,15].

ALKBH5 (AlkB homolog 5) is an m^6^A demethylase that is mainly localized in the nucleus and functions in RNA metabolism. It removes methyl groups from specific RNA transcripts and thereby influences cellular activity. When ALKBH5 expression or activity is reduced, global m^6^A levels increase, which can disturb normal cellular functions [16,17]. In metabolic diseases, ALKBH5 participates in the regulation of glucose and lipid metabolism by controlling the m^6^A status of key metabolic transcripts. Through these effects, ALKBH5 can influence insulin signaling, cellular responses to nutrient availability, and adaptation to metabolic stress. Changes in ALKBH5 expression have been observed in the liver, adipose tissue, and heart under conditions of obesity or insulin resistance, which suggests a potential role in therapeutic intervention. However, the effects of ALKBH5 differ between tissues, and its upstream regulation and downstream targets form complex networks. These features create challenges for translating current findings into clinical applications [18,19,20,21].

In this context, this review outlines the structural characteristics and molecular mechanisms of ALKBH5 and highlights its emerging roles in metabolic diseases, including MAFLD, diabetic complications such as diabetic retinopathy (DR), diabetes-associated cognitive impairment (DACI), atherosclerosis (AS), and diabetic cardiomyopathy (DCM), as well as metabolism-related inflammatory diseases such as rheumatoid arthritis (RA). Recent progress in pharmacological strategies targeting ALKBH5 is also summarized, together with challenges related to its tissue-specific regulatory functions. Improved understanding of ALKBH5-mediated epitranscriptomic regulation may provide new insights into the pathogenesis of metabolic diseases and support the development of novel therapeutic approaches.

## 2. Molecular Composition, Structural Features, and Catalytic Mechanism of ALKBH5

**Figure 1:**
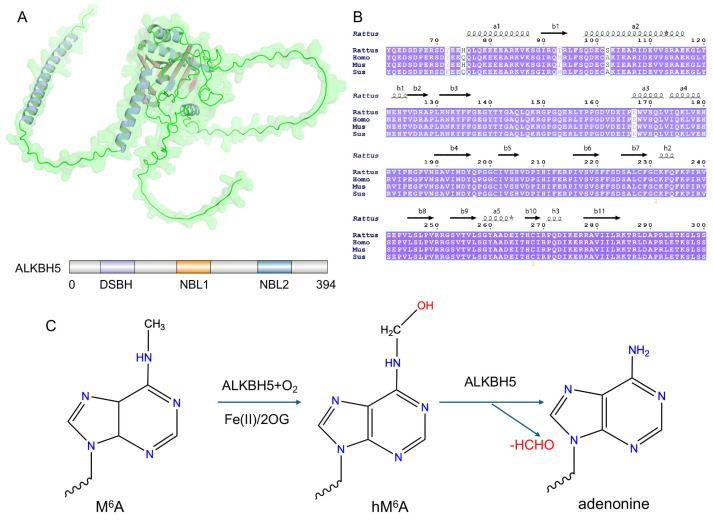
(**A**) The three-dimensional structure of ALKBH5 was predicted using AlphaFold 3 and visualized in PyMOL (version 2.5). The domain architecture and amino-acid sequence features were illustrated using IBS 2.0 [28,29,30]. (**B**) Conservation analysis of ALKBH5 protein performed with ESPript 3.2 [31]. (**C**) Mechanism of ALKBH5-mediated m^6^A demethylation: m^6^A is oxidized to a hydroxymethyladenosine (hm^6^A) intermediate and then demethylated to adenosine. Chemical structures were drawn with ChemDraw (version 22.0).

ALKBH5 is a member of the non-heme Fe(II)/2-oxoglutarate (2OG)-dependent dioxygenase family. These enzymes carry out oxidative changes on a range of biological substrates in vivo [17,22]. The human genome encodes nine AlkB homologs (ALKBH1–ALKBH8 and FTO). Among them, ALKBH5 and FTO act as the main m^6^A demethylases in mammalian cells. They function as epitranscriptomic “erasers” and thus change RNA metabolism and post-transcriptional gene expression in a dynamic way [23,24]. The ALKBH5 gene is located on chromosome 17p11.2. It encodes a protein of 394 amino acids with a mass near 43 kDa. The enzyme’s activity depends on a conserved catalytic core. That core coordinates cofactors, recognizes substrates, and supports the oxidative chemistry [16,25,26,27]. A schematic overview of the structural organization and catalytic mechanism of ALKBH5 is shown in Figure 1.

### 2.1. Catalytic Core Domain: DSBH Fold and Active Site

The catalytic core of ALKBH5 spans roughly residues 66–292 and adopts a conserved double-stranded β-helix (DSBH, also called a jelly roll) fold, a common feature of Fe(II)/2OG-dependent dioxygenases [32,33]. This fold is built from antiparallel β-strands arranged into a central eight-stranded β-sheet (some accounts label these strands β1–β11). Around this central sheet are α-helices that help to stabilize the overall structure. Together, these parts create a compact cavity that forms the enzyme’s active site. The DSBH scaffold provides the 3D framework needed for correct placement of the Fe(II) ion, the 2OG co-substrate, and the methylated RNA substrate. The scaffold pairs overall rigidity with local flexibility. This balance allows efficient oxygen activation, correct substrate positioning, and catalytic turnover [16,33].

#### 2.1.1. Fe(II) Coordination Site

Within the active-site cavity, Fe(II) binding depends on a conserved HX(D/E)X_n_H motif [34]. In ALKBH5, His204, Asp206, and His266 form this motif and directly coordinate the metal ion, thereby fixing it in a geometry suitable for catalysis. Correct positioning of Fe(II) is required for oxygen activation. It supports oxidative decarboxylation of 2OG and enables formation of the reactive iron–oxo intermediate that drives the demethylation reaction [22,35].

#### 2.1.2. Co-Substrate 2OG Binding Pocket

Binding of the 2OG co-substrate occurs near the Fe(II) center within the DSBH fold. This interaction relies on a network of polar and electrostatic contacts. Structural studies show that residues such as Asn193, Tyr195, Arg277, and Arg283 help stabilize 2OG and orient it for effective oxidative decarboxylation [17,36]. Variations in this local environment distinguish ALKBH5 from the related demethylase FTO. These differences may contribute to changes in reaction rate, co-substrate affinity, and substrate preference. Crystal structures also reveal sulfate ions near residues including Asn193 and Lys132. Such observations indicate that this region can interact with negatively charged groups and may influence substrate entry, local stability, or inhibitor binding [19,37,38].

#### 2.1.3. m^6^A Substrate Recognition and Binding Pocket

Recognition of the m^6^A modification occurs within a substrate-binding pocket located proximal to the catalytic core [39,40]. Several residues contribute to adenine stabilization, with Arg130 and Tyr139 playing central roles. The modified base inserts into the pocket and is held in place by hydrogen bonds and aromatic stacking interactions. The aromatic ring of Tyr139 is especially important, together with close contacts involving His204 [17,33,41]. This arrangement directs the N^6^-methyl group toward the Fe(II)/2OG center and places it within the proper distance for hydrogen abstraction. Through a precisely coordinated interaction network, ALKBH5 ensures high substrate selectivity and catalytic fidelity. Positively charged residues, such as Arg130, electrostatically stabilize the negatively charged RNA backbone, facilitating proper positioning within the catalytic pocket. Meanwhile, hydrogen bonding with the adenine base orients the N^6^-methyl group toward the Fe(II)/2-oxoglutarate catalytic center. In addition, π–π stacking interactions, particularly involving Tyr139, further stabilize the adenine ring and restrict its conformational flexibility. Collectively, these interactions precisely align the substrate for efficient hydrogen abstraction by the Fe(IV)=O intermediate while minimizing nonspecific oxidation [32,33].

### 2.2. Catalytic Mechanism of ALKBH5

The biological role of ALKBH5 is not limited to chemical removal of m^6^A modifications. Its activity affects RNA behavior at several levels, including RNA processing and cellular function. By removing m^6^A marks from selected transcripts, ALKBH5 changes RNA stability, alternative splicing patterns, translation efficiency, and transport between the nucleus and cytoplasm. These effects are linked to each other. As a result, single demethylation events can influence broader RNA regulatory networks and contribute to changes in cell state and disease-related processes [16,17]. Therefore, the importance of ALKBH5 lies in how its enzymatic activity is embedded in RNA regulatory systems rather than in the reaction alone.

At the biochemical level, ALKBH5 catalyzes m^6^A demethylation through a Fe(II)/2OG-dependent oxidative pathway that is typical for this enzyme family. Inside the catalytic pocket, Fe(II) binds 2OG and forms a complex that activates molecular oxygen. This step leads to oxidative decarboxylation of 2OG, which produces succinate and CO_2_. At the same time, a highly reactive ferryl–oxo intermediate (Fe(IV)=O) is generated. This intermediate serves as the key oxidizing species that links cofactor activation to modification of the RNA substrate and drives the demethylation reaction [17,22].

The Fe(IV)=O species removes a hydrogen atom from the N^6^-methyl group of m^6^A and forms a short-lived hydroxymethylated intermediate (hm^6^A). Unlike other Fe(II)/2OG-dependent demethylases, such as FTO, ALKBH5 uses a specific catalytic triad formed by Arg130, Lys132, and Tyr139. This triad rapidly captures hm^6^A and promotes its direct breakdown into adenosine and formaldehyde. This process limits release of the intermediate, supports efficient single-turnover demethylation, and maintains reaction accuracy under cellular conditions [16,23].

## 3. Mechanisms of ALKBH5 Action

**Figure 2:**
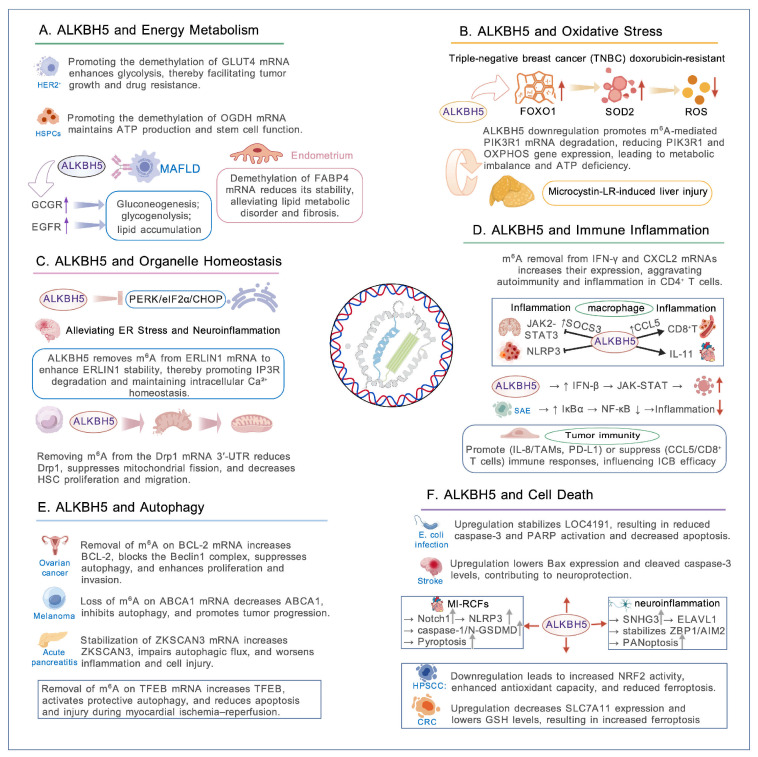
Mechanisms of ALKBH5 Action. (**A**–**F**) depict the relationships of ALKBH5 with energy metabolism, oxidative stress, organelle homeostasis, immune and inflammatory responses, autophagy, and cell death, respectively.

By removing m^6^A marks from selected mRNAs, ALKBH5 changes transcript stability and translation efficiency, and so affects key cellular processes such as energy metabolism, oxidative stress, organelle homeostasis, immune–inflammatory responses, autophagy, and cell death pathways including apoptosis, pyroptosis, and PANoptosis, as illustrated in Figure 2 [42,43,44,45].

### 3.1. ALKBH5 and Energy Metabolism

Cellular energy production mainly depends on two metabolic modes. Under aerobic conditions, cells generate ATP through the tricarboxylic acid (TCA) cycle and oxidative phosphorylation (OXPHOS). Under hypoxia or during rapid cell growth, cells rely more on glycolysis, a metabolic shift often described as the Warburg effect [46,47,48,49]. ALKBH5 influences the balance between these pathways, and its effects vary across cell types and physiological states.

ALKBH5 expression is markedly elevated in human epidermal growth factor receptor 2 (HER2)-positive breast cancer. Mechanistically, ALKBH5 promotes glycolytic reprogramming by removing m^6^A modification from glucose transporter 4 (GLUT4) mRNA. This demethylation reduces YTHDF2-dependent mRNA decay, thereby stabilizing GLUT4 transcripts and increasing protein expression. Elevated GLUT4 levels enhance glucose uptake and glycolytic flux, supporting cellular energy supply and biosynthetic demands. This mechanism, identified in HER2-positive breast cancer, also suggests a potential link between ALKBH5-mediated metabolic regulation and therapeutic response to HER2-targeted agents such as trastuzumab and lapatinib [50,51]. In contrast, ALKBH5 supports oxidative metabolism in hematopoietic stem and progenitor cells (HSPCs). By demethylating 2-oxoglutarate dehydrogenase (OGDH) mRNA, ALKBH5 maintains transcript stability and sustains TCA cycle function, mitochondrial activity, and ATP production. Loss of ALKBH5 accelerates OGDH mRNA degradation, weakens TCA cycle flux, lowers ATP levels, and reduces HSPC survival and functional capacity [52,53]. ALKBH5 also participates in the development of metabolic diseases. Elevated ALKBH5 expression has been observed in the livers of patients with obesity and T2DM. In mouse models, hepatocyte-specific deletion of Alkbh5 under high-fat diet conditions leads to reduced weight gain, improved glucose control, and less hepatic lipid accumulation. These results suggest a pathogenic role for ALKBH5 in metabolic imbalance. Mechanistically, ALKBH5 stabilizes glucagon receptor (GCGR) mRNA through m^6^A demethylation, which enhances glucagon signaling and increases hepatic glucose output, thereby worsening hyperglycemia. At the same time, ALKBH5 acts as a transcriptional co-regulator to increase epidermal growth factor receptor (EGFR) expression and activate mTORC1 signaling. This pathway promotes de novo lipid synthesis and lipid accumulation in the liver, contributing to hepatic steatosis [21,54]. Beyond the liver, ALKBH5-mediated metabolic regulation extends to fibrotic tissues. Beyond metabolic organs, ALKBH5 also affects energy metabolism in fibrotic tissues. Reduced ALKBH5 expression has been reported in samples from patients with endometrial fibrosis and intrauterine adhesion (IUA). Restoring ALKBH5 expression limits TGF-β1-induced fibrosis in endometrial cells by demethylating fatty acid binding protein 4 (FABP4) mRNA. This modification weakens IGF2BP2 binding, speeds up mRNA degradation, and lowers FABP4 protein levels. In rat models of IUA, ALKBH5 overexpression partially corrects abnormal lipid metabolic profiles, supporting tissue repair and reducing fibrosis through regulation of FABP4-dependent lipid metabolism [55,56].

Taken together, the effects of ALKBH5 on cellular metabolism may be related to cell type, the metabolic and inflammatory environment, and the repertoire of m^6^A-modified transcripts and their reader proteins. These factors may help explain why ALKBH5 is associated with maintenance of oxidative metabolism and cellular fitness in reparative settings, yet with glycolytic reprogramming, enhanced hormonal signaling, and lipid accumulation in cancer and metabolic disease. Understanding how these factors influence ALKBH5 function may inform more selective, tissue- and substrate-targeted strategies for therapeutic modulation.

### 3.2. ALKBH5 and Oxidative Stress

Oxidative stress occurs when cells make more reactive oxygen species (ROS) than they can remove, so ROS build up and damage DNA, proteins, and lipids, and harm cellular structure and genome integrity [57,58,59]. Emerging data show that ALKBH5 plays a key role in oxidative stress responses and in DNA repair, and that its m^6^A demethylase activity is modulated by ROS-dependent feedback [43].

Under oxidative stress, mitogen-activated protein kinase (MAPK) signaling is rapidly engaged, particularly via ERK1/2 and JNK pathways [60,61]. Phosphorylation of ALKBH5 by ERK/JNK strengthens its binding to the SUMO E2 enzyme UBC9 and promotes SUMOylation via the E3 ligase PIAS4. SUMO-1 attaches at K86 and K321. Together, phosphorylation and SUMOylation change ALKBH5 conformation and lower its activity by limiting RNA access to the catalytic center rather than by altering the active site directly [43,62,63].

ALKBH5 effects on oxidative stress depend on context. In doxorubicin-resistant triple-negative breast cancer (TNBC), ALKBH5 levels rise and the enzyme demethylates FOXO1 mRNA. This stabilizes FOXO1 transcript and raises its translation. Increased FOXO1 leads to higher SOD2 expression, lower intracellular ROS, and improved cell survival and drug resistance, forming an ALKBH5–FOXO1–SOD2 adaptive axis [64].

By contrast, microcystin-LR exposure reduces ALKBH5 in the liver. Higher m^6^A on PIK3R1 mRNA then promotes YTHDF3-mediated decay, which lowers PIK3R1 levels. Hepatocytes shift toward glycolysis and reduce expression of OXPHOS-related genes such as ETFDH, ETFA, and NDUFAF4. The result is mitochondrial dysfunction, broken energy balance, and less ATP [65].

ALKBH5 modulates oxidative stress responses in a manner that reflects the combined influence of cell type, the local metabolic and inflammatory environment, and post-translational modifications such as phosphorylation and SUMOylation. In some settings, these mechanisms enable ALKBH5 to stabilize transcripts like FOXO1, enhance antioxidant defenses, and preserve cellular energy, whereas in others, reduced ALKBH5 or altered m^6^A regulation contributes to mitochondrial dysfunction, impaired OXPHOS, and energy imbalance. Understanding how these factors interact to shape ALKBH5 activity and its target selection may provide a foundation for strategies aimed at selectively reinforcing its protective functions while limiting detrimental effects in vulnerable tissues.

### 3.3. ALKBH5 and Organelle Homeostasis

ALKBH5 acts beyond the nucleus and helps coordinate communication between organelles, especially the endoplasmic reticulum (ER) and mitochondria. In animal models of ischemic stroke, brain tissue shows marked upregulation of ALKBH5, suggesting an adaptive response to ischemic injury [66].

Mechanistic studies link ALKBH5 to STAT5 mRNA regulation. ALKBH5 removes m^6^A marks from STAT5 transcripts, and in the presence of the reader YTHDF1 this leads to lower STAT5 mRNA stability and reduced translation. Lower STAT5 protein levels blunt PERK pathway activation. As a result, PERK–eIF2α–CHOP signaling is damped, ER stress falls, and neuroinflammatory injury is reduced; conversely, ALKBH5 knockdown increases pathway activation and worsens cell damage [67].

ALKBH5 also controls ER–mitochondrial coupling via the ERLIN1–IP3R axis. Under normal conditions, ALKBH5 demethylates ERLIN1 mRNA, which raises ERLIN1 transcript stability and protein abundance. ERLIN1 promotes ER-associated degradation (ERAD) of activated inositol 1,4,5-trisphosphate receptors (IP3Rs), so IP3R levels remain controlled and calcium release from the ER is regulated. When ALKBH5 is lost or inhibited, ERLIN1 m^6^A rises, ERLIN1 transcripts fall, and ERLIN1 protein drops. That leads to IP3R accumulation, excessive ER calcium release, mitochondrial calcium overload, loss of membrane potential, and reduced energy production [44].

In liver fibrosis, ALKBH5 shapes mitochondrial dynamics in hepatic stellate cells (HSCs). Reduced ALKBH5 expression correlates with increased mitochondrial fission. At the molecular level, ALKBH5 removes m^6^A marks in the 3′-UTR of Drp1 mRNA and thereby lowers Drp1 translation in a YTHDF1-independent way. Loss of ALKBH5 raises Drp1 m^6^A and protein levels, which drives mitochondrial fragmentation and promotes HSC proliferation and migration [68]. This epitranscriptomic control of mitochondrial fission illustrates how ALKBH5 integrates organelle architecture, energy metabolism, and stress responses.

Overall, ALKBH5 may influence organelle homeostasis through regulation of ER and mitochondrial function, including m^6^A-dependent modulation of STAT5, ERLIN1, and Drp1 transcripts. These actions may help maintain ER–mitochondrial communication, calcium balance, and mitochondrial dynamics, supporting energy production and limiting cellular stress. Conversely, reduced or altered ALKBH5 may contribute to excessive ER calcium release, mitochondrial dysfunction, and pathological remodeling in liver fibrosis and ischemic injury. Understanding how ALKBH5 regulates these organelle-specific targets may provide insights for developing interventions that preserve cellular integrity while minimizing potential adverse effects.

### 3.4. ALKBH5 and Immune-Inflammatory Regulation

Metabolic diseases are characterized by chronic low-grade inflammation driven by dysregulated immune responses, which contribute to insulin resistance, metabolic imbalance, and tissue injury. Autoimmune diseases are driven by abnormal immune responses against self-tissues, which lead to persistent inflammation and organ damage. CD4^+^ T cells, especially Th1 and Th17 subsets, play a central role in these processes [69,70,71]. Recent studies show that ALKBH5 enhances the pathogenic activity of CD4^+^ T cells. In experimental autoimmune encephalomyelitis (EAE), ALKBH5 removes m^6^A marks from IFN-γ and CXCL2 mRNAs, thereby increasing their stability and translation. Elevated IFN-γ intensifies inflammatory responses, while CXCL2 promotes neutrophil recruitment and tissue infiltration, exacerbating inflammation and tissue injury. These mechanisms contribute not only to autoimmune pathology but also to chronic inflammation associated with metabolic dysfunction. Deletion of ALKBH5 specifically in T cells significantly reduces EAE severity, supporting a functional role for ALKBH5 in immune-mediated pathology [45,72,73].

ALKBH5 also participates in innate antiviral immune responses. During infection with DNA viruses such as HSV-1, KSHV, and MPXV, ALKBH5 undergoes lactylation, a modification that enhances its antiviral activity. Lactylated ALKBH5 selectively binds IFN-β mRNA, removes m^6^A modification, and increases transcript stability and translation. Enhanced IFN-β production activates JAK–STAT signaling and induces interferon-stimulated genes, leading to antiviral protection. Sustained activation of this pathway may also influence metabolic processes, including mitochondrial function and lipid metabolism [74].

In neuroinflammatory conditions, including traumatic brain injury (TBI), ALKBH5 acts as a regulator that limits excessive inflammation. After TBI, ALKBH5 expression increases in microglia and macrophages. ALKBH5 demethylates Socs3 mRNA, which stabilizes the transcript and increases SOCS3 protein levels. SOCS3 suppresses excessive JAK2–STAT3 signaling, restrains microglial overactivation, and reduces prolonged neuroinflammation. However, persistent SOCS3 activation has been linked to impaired insulin signaling. Loss of ALKBH5 produces the opposite effect and aggravates tissue injury [75].

In other disease settings, ALKBH5 can promote inflammation. In atherosclerosis, macrophages within plaques show increased ALKBH5 expression. ALKBH5 demethylates CCL5 mRNA, which increases its stability and activates CCR5 signaling. This process promotes foam cell dysfunction and recruitment of CD8^+^IFNγ^+^ T cells, thereby intensifying local inflammation and accelerating plaque progression [76].

A similar pattern is observed in cardiac fibrosis, where ALKBH5 stabilizes IL-11 mRNA and promotes macrophage-to-myofibroblast transition. In contrast, in metabolic disorders such as glucose-6-phosphate transporter (G6PT) deficiency, lactate-induced ALKBH5 demethylates NLRP3 mRNA and suppresses inflammasome activation [77,78]. In bacterial product-driven inflammation, such as sepsis-associated encephalopathy, lipopolysaccharide (LPS) reduces ALKBH5 expression. This increases m^6^A levels and enhances NF-κB signaling, while restoration of ALKBH5 stabilizes NFKBIA mRNA and limits pro-inflammatory gene expression [79].

Within the tumor immune microenvironment, ALKBH5 exerts divergent effects across different cancer types. In glioblastoma, hypoxia-induced ALKBH5 upregulates IL-8 expression, thereby promoting the recruitment of immunosuppressive tumor-associated macrophages and facilitating immune evasion. In intrahepatic cholangiocarcinoma, ALKBH5 increases PD-L1 expression via a YTHDF2-dependent mechanism, leading to impaired T cell-mediated cytotoxicity. In contrast, in colorectal cancer, elevated ALKBH5 expression is associated with enhanced CD8^+^ T cell infiltration and improved patient outcomes, potentially through CCL5-mediated recruitment of effector T cells [80]. ALKBH5 also influences responses to immune checkpoint therapy. Tumor-specific deletion of ALKBH5 enhances anti-PD-1 efficacy by altering lactate metabolism, reducing regulatory T cells and myeloid-derived suppressor cells, and reshaping the immunosuppressive environment. Clinical data further show that low ALKBH5 expression or loss-of-function mutations correlate with better responses to PD-1 blockade [81].

Overall, ALKBH5 may exert bidirectional effects on immune and inflammatory regulation by modulating the stability and translation of key transcripts, including cytokines, chemokines, and inflammasome components. These mechanisms can link epitranscriptomic control to both chronic inflammation in metabolic disorders and immune-mediated tissue injury, while altered ALKBH5 activity may either amplify or restrain inflammatory responses depending on the cell type, local microenvironment, and specific m^6^A targets. Elucidating how these factors shape ALKBH5 function could provide insights for selectively modulating immune and inflammatory pathways in disease settings.

### 3.5. ALKBH5 and Autophagy

Autophagy is a fundamental cellular process that preserves intracellular homeostasis by removing damaged organelles, misfolded proteins, and metabolic byproducts via the autophagosome–lysosome pathway, thereby restoring metabolic balance and enabling adaptive responses [82,83,84]. As an m^6^A-dependent post-transcriptional regulator, ALKBH5 modulates autophagy in a strongly context-dependent manner. Its effects vary with cell type, disease state, and the function of specific downstream targets [44,85].

ALKBH5 suppresses autophagy by regulating m^6^A modification of autophagy-related transcripts, thereby influencing cellular stress responses. In epithelial ovarian cancer, ALKBH5 overexpression demethylates BCL-2 mRNA, increasing transcript stability and protein abundance. Elevated BCL-2 disrupts formation of the Beclin1–PIK3C3 complex, blocking autophagy initiation. At the same time, activation of the EGFR–PI3K–AKT–mTOR pathway further reinforces autophagy inhibition, supporting tumor cell survival, proliferation, and invasion [86]. In cutaneous melanoma, ALKBH5 demethylates ABCA1 mRNA and reduces its stability, which suppresses autophagy and enhances metastatic potential [87]. In acute pancreatitis, ALKBH5 stabilizes ZKSCAN3 mRNA, a transcriptional repressor of autophagy-related genes. Increased ZKSCAN3 nuclear localization broadly inhibits autophagic flux, aggravating acinar cell damage and inflammation [88].

In contrast, ALKBH5 can also activate protective autophagy under specific conditions. During myocardial ischemia–reperfusion injury, ALKBH5 counteracts METTL3-mediated methylation of TFEB mRNA, increasing TFEB stability. TFEB acts as a central regulator of autophagy and lysosome biogenesis and, in turn, transcriptionally upregulates ALKBH5 while suppressing METTL3, forming a negative feedback loop. Under hypoxia–reoxygenation stress, this ALKBH5–TFEB axis enhances autophagic clearance of damaged cellular components, limits cardiomyocyte apoptosis, and provides cardioprotection [89].

Overall, ALKBH5 may influence autophagy by modulating the m^6^A status of key transcripts, including BCL-2, ABCA1, ZKSCAN3, and TFEB, thereby integrating signals related to metabolism, inflammation, and cellular stress. Depending on the specific target and cellular environment, ALKBH5 may either suppress or enhance autophagic activity, which can contribute to disease progression in cancers and inflammatory conditions or provide protection in stress-related injuries such as myocardial ischemia–reperfusion. A clearer understanding of these target- and tissue-specific effects may help guide strategies to modulate autophagy without inadvertently worsening pathology.

### 3.6. ALKBH5 and Multilayered Regulation of Cell Death

When cellular stress exceeds adaptive capacity, cells undergo diverse forms of regulated death—including apoptosis, necroptosis, pyroptosis, ferroptosis, cuproptosis, and autophagic cell death—each governed by distinct molecular circuits [90,91,92]. ALKBH5 does not function as a universal pro-survival or pro-death factor. Instead, it acts as a context-dependent regulator of cell fate by selectively demethylating m^6^A-modified mRNAs in different cell types and disease settings.

Under some conditions, ALKBH5 is cytoprotective. In *Escherichia coli* infection models, ALKBH5 stabilizes the lncRNA LOC4191 and suppresses activation of the caspase-3–PARP apoptotic pathway [93]. In ischemic stroke models, including in vivo MCAO and in vitro OGD/R, ALKBH5 expression is increased as a compensatory response. Knockdown of ALKBH5 enhances neuronal apoptosis, accompanied by increased Bax and cleaved caspase-3 levels, thereby aggravating brain injury [67].

ALKBH5 also regulates inflammatory forms of cell death. In myocardial infarction, ALKBH5 is upregulated in cardiac fibroblasts and demethylates Notch1 mRNA, which promotes NLRP3 inflammasome activation. This leads to caspase-1 cleavage and N-GSDMD-dependent pyroptosis. Inhibition of ALKBH5 reduces fibroblast pyroptosis and improves cardiac function [94]. ALKBH5 further participates in PANoptosis, a coordinated cell death program integrating pyroptosis, apoptosis, and necroptosis. Suppression of the ALKBH5/m^6^A/SNHG3 axis destabilizes the SNHG3–ELAVL1–ZBP1/AIM2 complex, reduces apoptosis-related gene expression, and alleviates inflammatory injury [95].

Ferroptosis is similarly context-dependent. In hypopharyngeal squamous cell carcinoma, ALKBH5 downregulation increases m^6^A on NFE2L2 (NRF2) mRNA, allowing IGF2BP2-mediated stabilization, enhancing antioxidant defenses, and preventing lipid peroxidation [96]. In contrast, in colorectal cancer, ALKBH5 overexpression demethylates SLC7A11 mRNA, decreases its stability and protein levels, reduces glutathione synthesis, and sensitizes cells to ferroptotic death [97].

Overall, ALKBH5 may influence diverse forms of regulated cell death by modulating the m^6^A status of specific transcripts, including LOC4191, Notch1, SNHG3, NFE2L2, and SLC7A11. Depending on the cell type, the nature of stress, and the affected targets, ALKBH5 activity can reduce apoptosis in neurons, limit fibroblast pyroptosis, modulate inflammatory PANoptosis, or alter susceptibility to ferroptosis in cancer cells. These findings suggest that precise understanding of target-specific and stress-specific effects is critical for developing therapeutic strategies that leverage ALKBH5’s regulatory potential while avoiding unintended promotion of tissue injury or disease progression.

## 4. ALKBH5 and Metabolic Diseases

**Figure 3:**
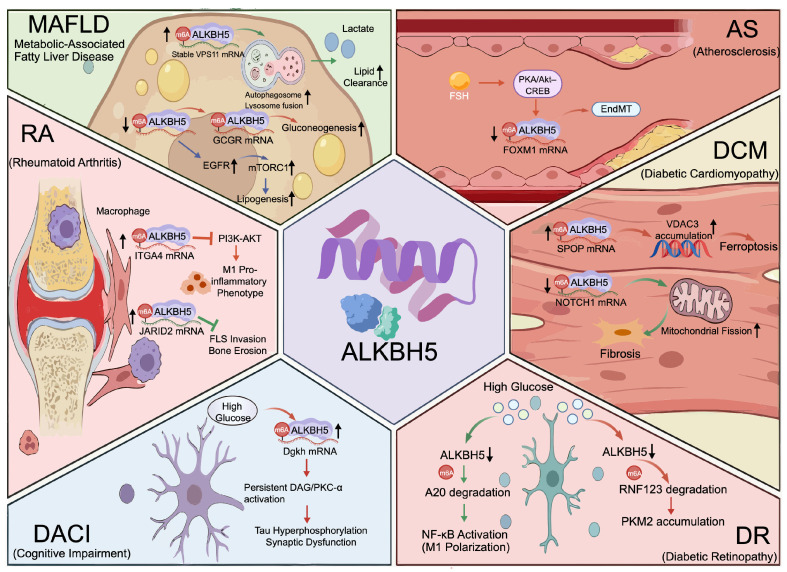
ALKBH5 and metabolic diseases. ALKBH5 influences metabolic diseases by regulating RNA processing and gene expression programs related to metabolic homeostasis. The diseases shown in this figure are categorized into three groups: MAFLD; metabolic complications including DR, DACI, AS, and DCM; and metabolism-related inflammatory diseases, represented by RA.

Metabolic diseases are characterized by disrupted energy metabolism and homeostasis, including diabetes, obesity, MAFLD, hyperlipidemia, and metabolic syndrome. Their pathogenesis involves insulin dysfunction, impaired glucose and lipid metabolism, energy imbalance, and chronic inflammation, leading to multi-organ damage and cardiometabolic complications [98,99,100]. As shown in Figure 3, ALKBH5 influences metabolic diseases by regulating RNA processing and expression programs related to metabolism, apoptosis, inflammation, and fibrosis.

### 4.1. ALKBH5 and Metabolic-Associated Fatty Liver Disease

MAFLD is a common diabetic complication, characterized by excessive hepatic lipid accumulation and aggravated insulin resistance [101,102]. In this setting, ALKBH5 acts in a context-dependent way: it can help maintain lipid balance in the liver by supporting autophagy, but it can also worsen glucose and lipid problems when metabolic stress is present.

Early work linked ALKBH5 to autophagic function. In HFD models and in patient samples, loss of ALKBH5 impaired autophagosome–lysosome fusion and reduced lipid clearance. By contrast, ALKBH5 overexpression increased VPS11 translation through m^6^A demethylation, boosted autophagic flux, and promoted lipid breakdown, pointing to a protective role for ALKBH5 [85].

However, obesity and HFD often cause strong upregulation of hepatic ALKBH5, and removing Alkbh5 in hepatocytes under HFD reduces weight gain, improves blood glucose, and lessens steatosis. Mechanistic studies indicate two routes. In an m^6^A-dependent route, ALKBH5 demethylates GCGR mRNA, stabilizes the transcript and protein, and therefore amplifies glucagon signaling to raise gluconeogenesis and glycogenolysis. In an m^6^A-independent route, ALKBH5 acts at an EGFR enhancer as a transcriptional co-regulator, activates mTORC1 signaling, and drives de novo lipogenesis, which promotes lipid build-up in the liver [21,54].

These observations position ALKBH5 as a nodal integrator of hormonal and growth factor signaling in the liver. Its dual regulatory capacity allows coordinated control of glucose and lipid metabolism, yet also underlies paradoxical effects under metabolic stress, highlighting that therapeutic targeting of ALKBH5 requires careful consideration of temporal, cellular, and disease-stage specificity to avoid unintended exacerbation of metabolic dysfunction.

### 4.2. ALKBH5 and Atherosclerosis

AS is a progressive, metabolism-associated vascular disorder driven by chronic inflammation and lipid deposition. Its initiation and progression are closely linked to endothelial dysfunction, dysregulated lipid metabolism, and sustained immune–inflammatory activation [103,104,105]. In conditions such as diabetes, obesity, and metabolic syndrome, vascular endothelial cells face high glucose, excess lipids, and oxidative stress. These insults break endothelial balance, bring in more monocytes and macrophages, and push cells toward a pro-inflammatory state, which speeds plaque growth and makes plaques less stable [105,106].

ALKBH5 exhibits cell type-specific roles in atherosclerosis. In postmenopausal women, elevated follicle-stimulating hormone (FSH) activates ALKBH5 via the PKA/Akt–CREB pathway. Under these conditions, ALKBH5 stabilizes FOXM1 mRNA in a HuR-dependent manner, increases SNAIL expression, and drives endothelial-to-mesenchymal transition (EndMT), a change that may weaken plaques and promote instability [107].

These findings highlight that therapeutic strategies targeting ALKBH5 in atherosclerosis should be carefully tailored to specific cellular contexts. In particular, modulation of ALKBH5 in endothelial cells warrants caution, as its activation, especially under hormone-related signaling such as FSH, may promote EndMT and contribute to plaque instability through the HuR–FOXM1–SNAIL axis.

### 4.3. ALKBH5 in Diabetic Cardiomyopathy

DCM constitutes a critical pathological basis for heart failure and increased mortality in patients with metabolic disorders. Its progression involves multiple forms of cell death, including apoptosis and ferroptosis, and accumulating evidence indicates that ALKBH5 exerts context-dependent roles in regulating these processes [108,109,110,111].

In diabetic cardiac fibrosis models, ALKBH5 expression is reduced. Loss of ALKBH5 increases m^6^A modification of NOTCH1 mRNA, which is then recognized by the reader YTHDF2 and degraded. Reduced NOTCH1 levels promote mitochondrial fission and enhance cardiac fibroblast proliferation, which worsens fibrotic remodeling. In contrast, ALKBH5 overexpression stabilizes NOTCH1 mRNA, suppresses mitochondrial fission, and limits fibroblast expansion, indicating a protective role against cardiac fibrosis [112].

In contrast, in DCM models and cardiomyocytes exposed to high glucose, ALKBH5 expression is increased and contributes to disease progression through ferroptosis. Under these conditions, ALKBH5 demethylates the 3′-UTR of speckle-type POZ protein (SPOP) mRNA. This modification blocks IGF2BP2-mediated stabilization and lowers SPOP levels. SPOP normally promotes ubiquitination and degradation of targets such as VDAC3. Reduced SPOP leads to VDAC3 accumulation, which triggers ferroptotic death of cardiomyocytes and accelerates DCM development [113].

These findings reveal that ALKBH5 exerts bidirectional, pathway-specific effects in diabetic cardiomyopathy. It can promote ferroptosis through the ALKBH5–SPOP–VDAC3 axis in some contexts, while in others, its reduction destabilizes cardioprotective transcripts and worsens disease. This duality highlights a fundamental challenge: indiscriminate modulation of ALKBH5 may produce opposing effects depending on disease stage and dominant molecular targets. The complexity of its context-dependent roles underscores the need for precise, target- and stage-specific strategies when considering ALKBH5 as a therapeutic target in DCM, as oversimplified interventions could inadvertently exacerbate cardiac injury.

### 4.4. ALKBH5 in Diabetic Retinopathy

DR is the most common microvascular complication of diabetes and a leading cause of vision loss. It features retinal microvascular damage, breakdown of the blood–retina barrier, and chronic neuroinflammation. Overactive retinal microglia are a main driver of disease progression [114,115,116].

High glucose reduces ALKBH5 expression in retinal microglia. Lower ALKBH5 raises m^6^A levels on A20 (TNFAIP3) mRNA and promotes YTHDF2-dependent decay. A20 normally restrains NF-κB signaling; loss of A20 removes that brake and sustains NF-κB activation. As a result, microglia shift to a pro-inflammatory M1 state and release more IL-1β, IL-6, and TNF-α, which maintain chronic inflammation and damage retinal neurons and blood vessels [117].

In Müller glial cells, hyperglycemia increases lactate levels and drives histone H3K18 lactylation, which upregulates ALKBH5. Higher ALKBH5 removes m^6^A from RNF123 mRNA and speeds its degradation. Reduced RNF123 lowers ubiquitination and clearance of pyruvate kinase M2 (PKM2), allowing PKM2 to accumulate. Elevated PKM2 enhances glycolysis, activates glia, and raises inflammatory cytokine release, thereby worsening retinal injury [118].

These findings reveal a striking cell type-specific regulation of ALKBH5 in DR. While its downregulation in microglia amplifies inflammatory and neurodegenerative responses, upregulation in Müller and RPE cells drives metabolic dysregulation and tissue injury. The opposing effects across retinal cell types highlight a fundamental challenge: therapeutic strategies targeting ALKBH5 must be precisely tailored to cell-specific contexts, as indiscriminate modulation risks worsening retinal pathology rather than alleviating it.

### 4.5. ALKBH5 in Diabetes-Associated Cognitive Impairment

DACI encompasses deficits in memory, executive function, and attention arising from chronic metabolic dysregulation, particularly sustained hyperglycemia and insulin resistance, and can progress to dementia. A hallmark pathological feature is abnormal hyperphosphorylation of the microtubule-associated protein Tau [119,120].

Recent studies identify ALKBH5 as an important regulator of DACI through an m^6^A-dependent pathway. Under high-glucose conditions, ALKBH5 expression is reduced in hippocampal neurons. This reduction increases m^6^A modification on diacylglycerol kinase η (Dgkh) mRNA and accelerates its degradation. Lower Dgkh levels lead to accumulation of diacylglycerol and sustained activation of protein kinase C-α (PKC-α). Continuous PKC-α signaling promotes Tau hyperphosphorylation, destabilizes microtubules, and induces abnormal Tau aggregation. These changes impair axonal transport and synaptic function and result in cognitive decline. In animal models, hippocampal overexpression of Dgkh reduces Tau pathology and improves cognitive performance, supporting a central role for the ALKBH5–Dgkh–PKC-α–Tau signaling axis in DACI [121].

These findings reveal a neuron-specific epitranscriptomic mechanism linking metabolic stress to Tau pathology and cognitive decline. However, the dualistic regulation of ALKBH5 across cell types and disease contexts raises a cautionary note: indiscriminate modulation of ALKBH5 could have opposing effects, potentially alleviating cognitive deficits in neurons while exacerbating metabolic or inflammatory dysregulation elsewhere, emphasizing the need for highly targeted, context-specific therapeutic strategies.

### 4.6. ALKBH5 and Rheumatoid Arthritis

RA is a chronic autoimmune disease marked by persistent synovial inflammation, progressive cartilage damage, and bone erosion. These pathological changes arise from coordinated actions of multiple cell types, mainly macrophages and fibroblast-like synoviocytes (FLSs) [122,123,124]. Notably, many of the cellular and molecular mechanisms implicated in RA, including macrophage polarization, fibroblast proliferation and invasion, and dysregulated tissue remodeling, are also perturbed in metabolic diseases such as diabetes, obesity, and MAFLD [125,126]. This overlap suggests that RA provides a valuable context for studying how ALKBH5-mediated m^6^A modifications influence immune and stromal cell behavior in metabolic inflammation. Recent studies show that the m^6^A demethylase ALKBH5 regulates these processes in a manner that depends on both cell type and local conditions.

In macrophages, increased expression of the piRNA piENOX2 accelerates degradation of Alkbh5 mRNA and lowers ALKBH5 protein levels. Reduced ALKBH5 leads to higher m^6^A modification on ITGA4 mRNA, which destabilizes the transcript and decreases ITGA4 protein expression. This change weakens PI3K–AKT signaling and shifts macrophages toward an M1 pro-inflammatory state, thereby intensifying synovial inflammation and joint injury [127].

In FLSs, especially under hypoxic conditions typical of inflamed joints, ALKBH5 expression is strongly increased. Higher ALKBH5 reduces m^6^A modification on JARID2 mRNA and weakens its binding to the m^6^A reader IGF2BP3. This lowers JARID2 expression and removes restraints on FLS proliferation and invasion, while also increasing the expression of several matrix metalloproteinases. In animal models, including collagen-induced arthritis and delayed-type hypersensitivity, local or systemic inhibition of ALKBH5 reduces joint swelling, synovial hyperplasia, cartilage destruction, and inflammatory cell infiltration, and is accompanied by recovery of JARID2 expression [128].

These findings establish ALKBH5 as a microenvironment-responsive regulator that modulates m^6^A in distinct cell populations, coordinating immune activation, stromal invasiveness, and tissue remodeling in RA. However, its dual and context-dependent functions suggest that indiscriminate targeting of ALKBH5 could yield opposing effects in different cell types, highlighting the need for precise, cell-specific therapeutic strategies. The relationship between ALKBH5 and metabolic diseases is summarized in Table 1.

## 5. Pharmacological Inhibitors Targeting ALKBH5

**Figure 4:**
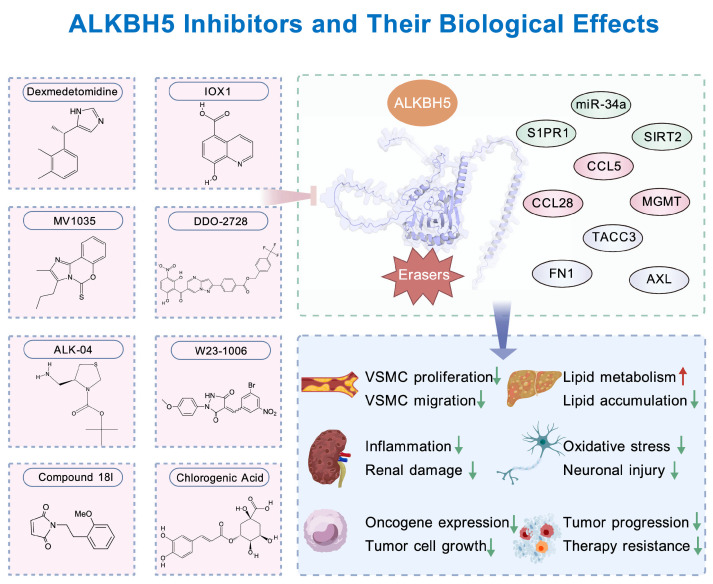
Pharmacological inhibitors targeting ALKBH5. ALKBH5 inhibitors, including Dexmedetomidine, IOX1, MV1035, DDO-2728, ALK-04, W23-1006, Compound 18L, and chlorogenic acid, suppress ALKBH5 activity, thereby modulating m^6^A demethylation and improving various diseases, such as metabolic-associated fatty liver disease, atherosclerosis, cancers, and leukemia.

In recent years, pharmacological strategies targeting ALKBH5 have expanded rapidly, encompassing drug repurposing, rationally optimized small molecules, and natural product discovery. Overall (Figure 4), these inhibitors elevate intracellular m^6^A levels, reshaping key post-transcriptional regulatory networks and modulating biological processes at multiple levels, including inflammation, oxidative stress, metabolic reprogramming, tumor cell plasticity, and the immune microenvironment. These advances provide novel avenues for epigenetic intervention in diverse disease contexts.

### 5.1. Clinical-Grade ALKBH5 Inhibitors

Dexmedetomidine, a clinically approved α_2_-adrenergic receptor agonist, has recently been identified as a functional inhibitor of ALKBH5, exerting biological effects beyond its established roles in sedation and analgesia [129,130]. In a HFD and streptozotocin (STZ)-induced rat model of diabetic peripheral neuropathy, dexmedetomidine reduces miR-34a expression, thereby relieving miR-34a-mediated repression of SIRT2 and suppressing S1PR1 transcription. This regulatory cascade ultimately attenuates oxidative stress, preserves mitochondrial integrity, and limits neuronal apoptosis [131]. Consistently, in lipopolysaccharide-induced tubular epithelial injury, dexmedetomidine downregulates ALKBH5 expression and recapitulates the anti-inflammatory and anti-apoptotic phenotypes observed upon genetic ALKBH5 knockdown [132]. Collectively, these findings indicate that dexmedetomidine modulates disease-relevant pathways not only through canonical α_2_-adrenergic signaling but also via post-transcriptional and epitranscriptomic mechanisms, particularly miRNA regulation and m^6^A demethylation. Importantly, these observations highlight the feasibility of pharmacologically targeting ALKBH5 using clinically deployable agents and underscore its therapeutic potential in metabolic and inflammatory diseases.

### 5.2. ALKBH5 Small-Molecule Inhibitors

IOX1 is a broad-spectrum inhibitor of 2-OG-dependent dioxygenases, targeting both JmjC-domain histone demethylases and select RNA demethylases, including ALKBH5, whose activity depends on the Fe(II)–2-OG catalytic system. In macrophages derived from human atherosclerotic plaques and in HFD-fed Apoe^−^/^−^ mice, ALKBH5 is upregulated in infiltrating macrophages, where it reduces m^6^A modification and stabilizes CCL5 mRNA. This enhances CCL5/CCR5-mediated autophagy and inflammatory signaling, thereby promoting senescent foam macrophage formation and accelerating plaque progression. Genetic deletion of ALKBH5, pharmacological inhibition with IOX1, or CCR5 blockade disrupts this pathogenic axis, leading to reduced inflammation and improved plaque stability [76]. Similarly, in kidney-specific Alkbh5 conditional knockout mice with renal ischemia–reperfusion injury, IOX1-mediated inhibition of ALKBH5 increases m^6^A modification and stability of CCL28 mRNA, enhances regulatory T cell recruitment, and alleviates inflammatory infiltration [133]. Despite these protective effects, the broad-spectrum activity of IOX1 raises concerns regarding selectivity and potential off-target toxicity, underscoring the need for further optimization in complex pathological contexts.

More selective ALKBH5 inhibitors have been developed primarily in oncology. MV1035, an imidazobenzoxazin-5-thione derivative, competitively inhibits ALKBH5 demethylase activity, elevates intracellular m^6^A levels, and suppresses pro-invasive gene expression. In glioblastoma models, MV1035 significantly inhibits migration and invasion of U87-MG cells and patient-derived glioblastoma stem cells and synergizes with temozolomide by suppressing MGMT-mediated DNA repair, thereby reducing tumor cell survival [134]. DDO-2728 is a selective ALKBH5 inhibitor, a pyrazolopyrimidine derivative (IC_50_ ≈ 2.97 μM), that suppresses ALKBH5-mediated m^6^A demethylation, thereby increasing m^6^A levels in AML cells, reducing TACC3 mRNA stability, inducing cell-cycle arrest, and exhibiting significant antitumor activity in a mouse MV4-11 xenograft model [135]. For example, ALK-04 combined with PD-1 blockade significantly prolongs survival in syngeneic tumor models using B16 melanoma in C57BL/6 mice and CT26 colorectal tumors in BALB/c mice, highlighting ALKBH5 as a potential immunoregulatory node within the epitranscriptomic metabolic immune axis [81].

Covalent ALKBH5 inhibitors exploit electrophilic warheads to form irreversible or highly efficient reversible bonds with the non-conserved cysteine residue Cys200 within the ALKBH5 active site, thereby achieving high specificity. W23-1006 suppresses triple negative breast cancer progression in MDA-MB-231 xenograft and lung metastasis models in immunodeficient female mice by enhancing m^6^A modification of FN1 mRNA, promoting its degradation and inhibiting proliferation, migration, and invasion [136]. Similarly, compound 18L, developed for leukemia, covalently binds Cys200 and potently inhibits ALKBH5 (in vitro IC_50_ = 0.62 μM; NB4 proliferation IC_50_ = 0.63 μM; K_d = 804 nM) without affecting FTO, and demonstrates robust antitumor efficacy in an NB4 xenograft model in female BALB/c nude mice [137]. These studies collectively illustrate that covalent inhibition enables precise modulation of ALKBH5 activity and m^6^A dynamics, offering effective intervention strategies for malignancies such as triple-negative breast cancer and leukemia.

### 5.3. Natural Product Inhibitors of ALKBH5

Natural products have emerged as an important source of ALKBH5 modulators, with an increasing number of small-molecule compounds reported to exhibit inhibitory activity. Compared with synthetic inhibitors, natural products often display advantages such as wide availability, favorable biocompatibility, and potentially lower toxicity [138,139]. Among these, chlorogenic acid (CGA), a widely distributed dietary polyphenol, has been shown to bind ALKBH5 and inhibit its demethylase activity. In a high-fat diet-induced MAFLD mouse model, oral administration of CGA significantly alleviates hepatic lipid accumulation, partly through enhancement of autophagy and suppression of MAPK/ERK signaling. Mechanistically, CGA reduces ALKBH5 activity, increases m^6^A modification of AXL mRNA, and downregulates AXL expression, thereby attenuating inflammatory responses and abnormal lipid metabolism [140,141].

These findings suggest that natural small molecules can modulate ALKBH5 activity, potentially through metal ion chelation or competitive binding. Future efforts may focus on systematic screening of plant-derived, dietary, or endogenous metabolites as pharmacological modulators of ALKBH5, with all reported inhibitors summarized in Table 2.

### 5.4. Targeted Protein Degradation (TPD)

TPD employs bifunctional molecules, such as PROTACs, to recruit target proteins to E3 ubiquitin ligases, thereby inducing selective ubiquitination and subsequent proteasomal degradation. Compared with conventional small-molecule inhibition, TPD offers a distinct advantage for proteins whose functions are difficult to fully suppress through catalytic inhibition alone [142,143]. Although no ALKBH5-directed degrader has been reported to date, ALKBH5 shares a conserved Fe(II)/2OG-dependent catalytic domain with its homolog FTO and plays essential roles in m^6^A regulation across a wide range of pathological contexts. Consequently, the development of FTO-targeted degraders provides a valuable conceptual and technical reference for future ALKBH5 degrader design [144]. Several FTO degraders, including QP73, FP54, and FTO-DT, have been generated by conjugating high-affinity FTO inhibitors with ligands recruiting either the CRBN or VHL E3 ubiquitin ligase. These bifunctional molecules efficiently induce FTO ubiquitination and proteasomal degradation and exhibit robust biological activity both in vitro and in vivo. Specifically, QP73 increases global m^6^A levels and suppresses oncogene expression, thereby inhibiting acute myeloid leukemia progression [145]; FP54 enhances m^6^A modification of ribosome biogenesis-related transcripts, promotes their YTHDF2-mediated decay, and restricts leukemia growth [146], and FTO-DT improves mitochondrial function and reduces lipid accumulation in metabolic disease models [147].

Collectively, these studies demonstrate the feasibility of degrading RNA demethylases and highlight key design principles for TPD strategies, including the selection of highly specific warheads, preferential recruitment of CRBN or VHL E3 ligases, and careful optimization of linker length and geometry to stabilize productive ternary complex formation. Although ALKBH5-targeted degraders remain to be developed, the established FTO-TPD framework provides a practical blueprint for constructing potent and selective ALKBH5 degraders in future studies.

## 6. Conclusions and Perspectives

As a central m^6^A RNA demethylase, ALKBH5 adds a dynamic epitranscriptomic layer to metabolic regulation. Through selective demethylation of mRNA substrates and interactions with regulatory proteins, it modulates transcript stability, translation, and fate, thereby coordinating glucose and lipid metabolism, insulin sensitivity, inflammatory responses, and cellular stress adaptation across tissues. Its functions are highly context- and cell type-dependent, reflecting the nuanced role of epitranscriptomic regulation in shaping metabolic disease heterogeneity.

However, ALKBH5 exhibits opposing effects depending on tissue, disease stage, and microenvironmental cues, complicating its therapeutic targeting. Its substrate spectrum remains incompletely defined, and most evidence stems from single-gene or pathway-focused studies that may not capture network-level effects. Emerging m^6^A-independent roles and potential long-term systemic consequences of RNA methylation manipulation remain poorly understood, emphasizing caution in extrapolating preclinical findings.

From a translational standpoint, ALKBH5 represents a conceptually novel intervention distinct from traditional receptor- or enzyme-based therapies. Yet, achieving tissue specificity, minimizing off-target epitranscriptomic effects, and identifying patient populations likely to benefit are significant challenges. Integrating single-cell and spatial epitranscriptomics with metabolic phenotyping and biomarker-guided stratification will be critical to define therapeutic windows and safety profiles.

Overall, ALKBH5 functions as a context-sensitive regulator rather than a universal metabolic switch. Understanding its spatiotemporal regulation and network-level impact is essential to translate epitranscriptomic insights into precise and safe interventions for metabolic disease.

## Figures and Tables

**Table 1:** Disease-associated alterations of ALKBH5 and underlying mechanisms.

Disease	Evidence Type	Target Cells/Tissues	ALKBH5 Change	Mechanism	Ref.
MAFLD	Human, Animal model, In vitro	Liver, Hepatocytes	Downregulated	Loss of ALKBH5 increases m^6^A modification of VPS11 mRNA, impairing autophagosome–lysosome fusion and lipid clearance	[85]
MAFLD	Animal model, In vitro	Liver, Hepatocytes	Upregulated	Demethylation of GCGR mRNA enhances glucagon signaling; ALKBH5 also acts as a transcriptional coregulator of EGFR–mTORC1 to promote lipogenesis	[54]
AS	Animal model, In vitro	Aorta, Vascular endothelial cells	Upregulated	Demethylation of FOXM1 mRNA in a HuR-dependent manner induces SNAIL expression and endothelial-to-mesenchymal transition	[107]
DCM	Animal model, In vitro	Heart, Cardiac fibroblasts	Downregulated	Enhanced m^6^A-mediated degradation of NOTCH1 mRNA via YTHDF2 promotes mitochondrial fission and fibrosis	[112]
DCM	Animal model, In vitro	Heart, Cardiomyocytes	Upregulated	Demethylation of the SPOP mRNA 3′UTR disrupts IGF2BP2-mediated stabilization, leading to VDAC3 accumulation and ferroptosis	[113]
DR	Human, Animal model, In vitro	Aetina, Retinal microglia	Downregulated	Increased m^6^A-dependent degradation of A20 mRNA sustains NF-κB activation and pro-inflammatory polarization	[117]
DR	Animal model, In vitro	Aetina, Müller glial cells	Upregulated	Demethylation-dependent destabilization of RNF123 mRNA causes PKM2 accumulation, glycolytic reprogramming, and inflammation	[118]
DACI	Animal model, In vitro	Hippocampal neurons, hippocampal neuron cell	Downregulated	m^6^A-dependent degradation of Dgkh mRNA activates DAG–PKCα signaling and promotes Tau hyperphosphorylation	[121]
RA	Human, Animal model, In vitro	Synovial tissue, Macrophages	Downregulated	Increased m^6^A on ITGA4 mRNA suppresses PI3K–AKT signaling and favors M1 polarization	[127]
RA	Human, Animal model, In vitro	Synovial tissue, Fibroblast-like synoviocytes	Upregulated	Reduced m^6^A on JARID2 mRNA lowers its expression, promoting FLS proliferation, invasion, and matrix metalloproteinase activity	[128]

**Table 2:** ALKBH5 Inhibitors and Their Biological Effects.

Type	Mechanism Type	Name	Disease	Model	Mechanism of Action	Ref.
Clinical Drug Inhibitor	Indirect	Dexmedetomidine	Diabetic Peripheral Neuropathy	HFD-induced rat, RSC96 cell	Inhibits ALKBH5, regulates miR-34a/SIRT2/S1PR1 signaling, reduces oxidative stress and cell apoptosis	[131]
Clinical Drug Inhibitor	Indirect	Dexmedetomidine	Acute Renal Tubular Inflammation	LPS-treated HK-2 cells	Inhibits ALKBH5 expression, exerts anti-inflammatory and anti-apoptotic effects	[132]
Small Molecule Inhibitor	Indirect	IOX1	Atherosclerosis	HFD-fed Apoe^−^/^−^ mice; human plaque-derived macrophages	ALKBH5 upregulation stabilizes CCL5 and drives plaque progression, while its loss, IOX1, or CCR5 blockade reverses this process.	[76]
Small Molecule Inhibitor	Indirect	IOX1	Renal Ischemia–Reperfusion Injury	Kidney-specific Alkbh5 conditional knockout mice	Inhibits ALKBH5, enhances m^6^A modification of CCL28 mRNA, promotes Treg recruitment and suppresses inflammatory infiltration	[133]
Small Molecule Inhibitor	Direct	MV1035	Glioblastoma	U87-MG cells; patient-derived GSCs	Competitive inhibition of ALKBH5, increases m^6^A modification, downregulates pro-invasion genes, suppresses migration and invasion	[134]
Small Molecule Inhibitor	Direct	DDO-2728	Acute Myeloid Leukemia	MV4-11 xenograft mouse model; AML cells	Increases m^6^A modification, decreases TACC3 mRNA stability, blocks cell cycle, inhibits tumor growth	[135]
Small Molecule Inhibitor	Direct	ALK-04	Tumor Immune Microenvironment Dysfunction	B16 melanoma (C57BL/6); CT26 colorectal tumor (BALB/c)	Inhibits ALKBH5, improves immune microenvironment, prolongs survival	[81]
Covalent Inhibitor	Direct	W23-1006	Triple-Negative Breast Cancer	MDA-MB-231 xenograft and lung metastasis models	Covalently binds ALKBH5 Cys200, enhances m^6^A modification of FN1 mRNA, promotes mRNA degradation, inhibits proliferation and invasion	[136]
Covalent Inhibitor	Direct	Compound 18L	Leukemia	NB4 xenograft (nude mice); NB4 cells	Covalently binds ALKBH5 Cys200, efficiently and selectively inhibits ALKBH5, suppresses cell proliferation and shows in vivo antitumor effect	[137]
Natural Product Inhibitor	Direct	CGA	MAFLD	HFD-induced MAFLD mouse model	Inhibits ALKBH5 demethylation, increases m^6^A modification of AXL mRNA, downregulates AXL expression, improves inflammation and lipid metabolism	[140]

## Data Availability

No new data were created or analyzed in this study.
